# St. John Long and His Disciples

**Published:** 1830-10-01

**Authors:** 


### LXIV.

### St. John Long.

When we closed our remarks on the tragedy of Miss Cashin, at page 528, the inquest had not terminated; but, in two days afterwards, the verdict of manslaughter was delivered by a jury of intelligent men, and after an investigation of unparalleled duration and minuteness, in which every kind of irrelevant matter was forced upon the inquisitors by the arts of legal sophistry, and the weakness, (to say the least of it) of the coroner. The storm which Mr. Wakley had to sustain from the defendant's counsel, would have overwhelmed most people of sensibility and modesty—but, for once, Mr. Adolphus met his match, and more than his match—for, although the subtle advocate, practiced and trained in forensic debate, ought to have been well aware that passion is a bad ingredient in argument, yet he repeatedly lost his temper, and with it the whole force of his ratiocination; while his opponent, though harrassed with every kind of personal allusion that could irritate the feelings, remained imperturbable, and consequently brought reason and truth to bear with irresistible impulse on the sophistry, and, in many points, the absurdity of his adversary's appeals to the jury. Thus Mr. Adolphus suffered his zeal or his excitement to completely eclipse his judgment, in the attempt to persuade any twelve men who were in their senses, and not under the delusion inspired by charlatanism, that the quack's liniment would only act on unsound parts of the body! Mr. Adolphus was, or appeared to be, blind to the sworn facts that Miss Cashin was in good health before the liniment was applied, and that all the organs of the body were found in a state of the most perfect integrity after death. Here his absurd theory was damned before his eyes, and yet he could not see the contradiction.

Upon the melancholy case itself we shall make no further remark, as the final adjudication is not yet made. We had some pity for St. John Long, till we learnt the heartless indifference with which he appears to view the horrible catastrophe! That heart, indeed, must be callous, which can reflect on the dire ignorance by which a beautiful young lady was sacrificed—not so much by the original application to the back, as by the reckless neglect of calling in surgical assistance, by which the effects of the burning liniment might have been readily obviated. But we fear that remorse has little to do with the profitable trade of quackery any where. Yet we imagine that the man who knows not how to manage the effects of his remedies, and obstinately persists in maltreating them till death closes the eyes of the deluded victim of ignorance and presumption, cannot sleep easy on his pillow. The still small voice of conscience will break through all the opiates of wealth, and corrode, unseen by the world, the most marble-hearted candidate for power or pelf.

Owing to the illness and absence of some of the most necessary witnesses, the Grand Jury did not proceed to the finding or ignoring the bill of indictment. It will therefore be six weeks before that preliminary step is taken. Meanwhile, Mr. Long follows his usual avocations, and the Aristocracy of this our enlightened land as firmly believe in the miraculous cures of a broken-down painter, as the disciples of Johannah Southcote believed in the advent of the young Shiloah! And why should not St. John Long extract from the pockets of the credulous fifty or sixty thousand pounds, when Mrs. Steevens sold some calcined egg's shells to Government for £10,000., as an infallible remedy for stone in the bladder?

For the honour of the profession, there is but one GULL (if he be not a DECOY-DUCK) who has come forward to bear witness to the miracles of the painter—Mr. Porter, commonly called Doctor among the *Niggers* on his estate in Jamaica. It is somewhat suspicious that this Octogenarian has taken up his *residence* in the house of the Charlatan. This advocate of Long was attended by Dr. Johnson some years ago, and, excepting the effects produced by the advance on Time's list, Dr. J. cannot perceive one particle of improvement in the state of his eyes—and as for the state of his lungs, he will carry chronic bronchitis to the tomb with him, as he has already carried it half his life-time, beneath the burning suns of the Antilles, the blue skies of Italy, and the cloudy atmosphere of Harley Street. Shame on Mr. Porter for abetting the delusions of a quack! There was but one medical man in Europe who would have done so—himself! By the way, why did not St. John Long take his medical decoy-duck to Miss Cashin, when the unfortunate lady's back was in such a dreadful state of excoriation? The ten thousand excoriated backs of slaves, which Surgeon Porter had ample opportunities of seeing in Jamaica, would have enabled him, on this point at least, to have offered his master, some wholesome advice! But—

Quem Deus vult perdere prius dementit.*

* St. John Long and his friends need not despair. We think there is great probability of his being acquitted of manslaughter, either by the grand or the petit jury; and, on mature consideration, we are inclined to think, that if the public are not to be enlightened by the disclosures at the coroner's inquest, they would not believe Miss Cashin, if she were to rise from the sepulchre! This being the case, a *condemnation* will only increase the zeal of his friends, who will represent the whole affair as a persecution, while an *acquittal* will enable the quack to boast of his triumphs! Mr. Wakley has done his duty manfully, and is deserving of praise for the trouble and expense already incurred—but we doubt whether any farther prosecution of the charlatan will be beneficial to the interests of society.

